# Bacterial Uropathogens, Antimicrobial Susceptibility Profile and Associated Factors among Pediatric Patients in Bahir Dar, Northwest Ethiopia

**DOI:** 10.4314/ejhs.v32i1.10

**Published:** 2022-01

**Authors:** Girma Zerefaw, Senait Tadesse, Awoke Derbie

**Affiliations:** 1 Liben Primary Hospital, West Gojjam, Ethiopia; 2 Department of Medical Microbiology, College of Medicine and Health Sciences, Bahir Dar University, Ethiopia; 3 Center for Innovative Drug development and Therapeutic Trial for Africa (CDT-Africa), Addis Ababa University, Ethiopia; 4 Biotechnology Research Institute, Bahir Dar University, Ethiopia

**Keywords:** Pediatric bacteriuria, urinary tract infection, antimicrobial resistance, Bahir Dar

## Abstract

**Background:**

Urinary tract infection (UTI) in the pediatric group may lead to end-stage renal dysfunction later in life. Tracking the type of the isolates and their antimicrobial resistance pattern would impact the management of UTI in these group. The aim of this study was to describe the distribution of bacterial uropathogenes, their antimicrobial susceptibility profile and factors associated with significant bacteriuria (SBU) among pediatric patients at selected facilities in Bahir Dar, Northwest Ethiopia.

**Methods:**

A cross-sectional study was conducted from 1^st^ February to 30^th^ June 2020. About 5–10ml of urine samples were collected from pediatrics presumptive for UTI and a urine sample was considered positive for SBU if a single organism was grown at a concentration of ≥10^4^cfu/ml. Antimicrobial sensitivity testing was performed using the Kirby-Bauer disc diffusion technique. Logistic regression was used to identify factors associated with SBU and statistical significance was set at p-value < 0.05.

**Results:**

Of the total 299 study participants, the majority 173 (57.9%) were females. The mean age of the participants was 6.6 years. The proportion of significant bacteriuria was at 49(16.4%). Most, 37 (75.5%) of the isolates were Gram-negative. The most predominant isolate was E. coli, 21(42.9%) followed by P. aeruginosa, 6(12.2%) and coagulase negative staphylococci, 6(12.2%). The level of multi-drug resistance among Gram-positive and Gram-negatives was at 50% and 78.4%, respectively. Participants' sex, circumcision status, having a flank pain and being malnourished were statistically associated with significant bacteriuria.

**Conclusion:**

Actions to minimize antimicrobial resistance should be strengthened to reduce the impact of UTI among the pediatric group.

## Introduction

Urinary tract infection (UTI) is an infection in any part of urinary system (kidneys, ureters, bladder and urethra) (1). It is one of the commonest bacterial infections encountered in daily clinical practice and a major problem that is frequently encountered by pediatrics healthcare providers (2, 3). It begins when uropathogens that reside in the gut contaminate the per-urethral area and /or through hematogenous spread, are able to colonize the urethra. Subsequent migration to the bladder and expression of different virulence factors results in colonization and invasion of the superficial umbrella cells (4).

Pediatrics urinary tract infection presentations are challenging because symptoms are vague and variable. It may present with poor feeding, fever and dysuria (5). The upper urinary tract infection involves the kidney parenchyma and may cause scarring and permanent damage that will proceed to cause hypertension and decreased kidney function (6).

Urinary tract infections are caused by both Gram-negative and Gram-positive bacteria. Among the agent of pathogens involved in UTI, *Escherichia coli* accounting for approximately 85% of the cases in children, followed by *Klebsiella pneumoniae, Proteus mirabilis*, *Enterococcus faecalis* and *Staphylococcus saprophyticus* (4).

The prevalence of pediatric UTI varies among countries due to geographical variation, antibiotic use pattern and other factors (7). An estimated 8% of girls and 2% of boys will have at least one episode by seven years of age. Of these children, 12–30% will experience recurrence within one year. For instance, the Australian hospital admission records indicate that pediatric UTIs represent 12% of all UTI hospital admissions (5). Renal parenchyma defects are present in 3 to 15% of children within one to two years of their diagnosis for UTI (8). Early diagnosis of pediatric UTI is necessary for prevention of renal damage and recurrence. Accurate diagnosis of UTI is essential to avoid any antibiotic overuse and expensive investigations (9).

The management of UTI is fraught with challenges which causes significant illness and is frequently missed, probably because of its non-specific presentation and similarity with other common illnesses especially in an era of increasing antimicrobial resistance (10, 11). Using the most appropriate antibiotic is essential to obtain the best results without compromising the patients' wellbeing. Supportive treatment like anti-pains together with non-pharmacological advice, such as good hygiene and adequate hydration, physiologic phimosis, circumcision, and education for adequate genital hygiene, breast feeding, and early toilet trained and voided dysfunctions are important to reduce the discomfort for the patient (12, 13).

The global spread of multidrug-resistant organisms has led to an increase UTIs in children that are difficult to treat (14). Antibiotic resistance in pediatrics is a global challenge in the public health sector and also a major challenge in Ethiopia as well (15).

Although there are a number of studies on bacteriology of UTI in Ethiopia, there is limited data on the field among the pediatric population, specifically, in the present study area. Every region in the country might have diversified population characteristics and it is important to know, especially, the antimicrobial resistance pattern of the isolates associated with UTI from different regions for an input to devise interventional strategies. Therefore, the aim of this study was to identify the type of bacterial uropathogens, their antimicrobial resistance profile and factors associated with significant bacteriuria among pediatric children at Felege Hiwot Comprehensive Specialized Hospital (FHCSH) and Sehate Birhan Specialized Pediatrics Clinic (SBSPC), northwest Ethiopia.

## Methods and Materials

**Study design, area and period**

A health facility based cross-sectional study was carried out at FHCSH and SBSPC, Bahir Dar in the period of 1^st^ February 2020 to 30^th^ Jun 2020. Bahir Dar is the capital city of Amhara Regional State situated about 565 kilometers to the Northwest of Addis Ababa, the capital city of Ethiopia. It is located 11.59 attitude and 37.39 longitudes (∼11°29′N latitude, 37°29′E longitude) and it is situated at elevation 1799 meters above sea level.

Felege Hiwot Comprehensive Specialized Hospital was established in 1952 and was serving more than 10 million people of Bahir Dar and the surrounding zones and regions. At the time of data collection, the Hospital had 13 wards, 430 beds, and about 531 health professionals. The outpatient clients were more than 24000 quarterly and daily outpatient clients were more than 600. The Pediatric clinic was one of the departments in the hospital in which about 1782 total pediatric children were attended quarterly and about 594 monthly and 21 daily for different health service (16).

**Population**: All pediatric patients whose age ranged between 0–18 years with suggestive symptom of UTI attending at FHCSH and SBSPC were considered as the source population while those with symptom for UTI in these facilities during the study period were the study population.

**Inclusion and exclusion criteria**: Pediatric patients whose age ranged between 0–18 years and presented with suggestive symptoms for UTI (such as fever, vomiting, and dysuria, urinary frequency more than the pervious/normal, urinary urgency as complained in older children and loin pain or urinary color change) were included in this study. In contrast those who had history of antibiotic usage two weeks prior to the data collection period were excluded.

While prevalence of significant bacteriuria, type of uropathogens, antimicrobial susceptibility profile of the isolates are dependent variables, age, sex, residence, educational status, previous history of UTI, history of catheterization, malnutrition, constipation, diabetes mellitus and circumcisions status (only male) of pediatric UTI patients were considered as independent variables.

**Sample size determination and sampling technique**: A single population proportion formula was used to calculate the sample size using the prevalence of significant bacteriuria among pediatric groups reported in Gondar, Ethiopia at (26.5) (17). Thus, the total sample size was at 299. This sample size was proportionally allocated for the two facilities and collected by convenient sampling technique.

**Data collection and processing**: After ensuring informed written consent from the parents or their guardians and assent from children, sociodemographic and clinical data were collected using structured questionnaires (some of the data were extracted from the patient card as well). Participant's nutritional status was measured using Mid-upper arm circumference (MUAC) by trained nurses and clinical examination was carried out by the attending physician. Accordingly, malnutrition was declared when MUAC reads below 13.

**Urine sample collection and bacteriological culture**: The guardians or patients were instructed how to collect a clean-catch midstream urine specimen. Accordingly, about 5–10 ml urine specimen was collected from each participant in a sterile screw-capped, widemouth container and labeled with the unique sample number, date and time of collection (18, 19). Using a cold box, all samples were transported to Bahir Dar University, College of Medicine and Health Science microbiology laboratory for bacteriological analysis.

Using calibrated wire loop (0.001 mL) urine samples were inoculated into Cystine Lysine Electrolyte Deficient medium (CLED). After incubation at 37°C for 24–48 hours, colonies were counted to check significant growth. Colony counts yielding bacterial growth of ≥10^4^ CFU/mL were regarded as significant for bacteriuria (SBU). Colonies from CLED were sub-cultured into MacConkey and blood agar plates (BAP) (Oxoid) and incubated at 37°C for 24–48 hours. Identification of bacteria were done using colony characteristics, Gram reaction and panel of biochemical tests. The Gram-negative bacteria were identified by indole and H^2^S production in lysine iron agar (LIA), citrate utilization, urease test, motility test, and oxides and carbohydrate utilization tests. The Gram-positive bacteria were identified using catalase, coagulase and bilesculin test (18, 19).

**Antimicrobial susceptibility testing (AST)**: The antimicrobial resistance profiles of all identified bacterial isolates were performed *in vitro* according to the criteria of Clinical and Laboratory Standards Institute (CLSI) using the Kirby-Bauer disc diffusion method. A loop full of bacteria (3–5 identical colonies) were taken from a pure culture colony and transferred to a tube containing 5 ml of normal saline and mixed gently until it forms a homogenous suspension. The turbidity of the suspension adjusted to the turbidity of McFarland 0.5 in order to standardize the inoculums size and swabbed on Muller Hinton medium using a sterile cotton swab.

The following antimicrobial discs with their respective concentration were used: meropnem [10µg], ceftazidime [30 µg], ceftriaxone (30µg), penicillin [10 µg], nitrofurantoin [30 µg], gentamycin [10µg], ampicillin (5µg), amoxicillin-clavulnate [30 µg], ciprofloxacin (5µg), trimethoprim-sulphamethaxzole [1.25/23.75 µg], and nialdxic acid [30 µg] for Gram-negative bacteria, whereas nitrofarantin [30µg], erythromycin [15µg], ciprofloxacin (5µg), trimethoprime/sulfamethoxazole [1.25/23.75 µg], chloramphenicol [30µg], clindamicin [30 µg], amoxicillin-clavulnate [30 µg], penicillin [100 µg], oxaclin [1µ g], ampicillin (5µg) and vancomicin [30 µg], for Gram-positive isolates. The criteria used to select the antimicrobial agents were based on both their availability for the management of UTIs in the study area and CLSI guideline. The plates were incubated at 37oC for 24 hours. Diameters of the zone of inhibition around the discs were measured to the nearest millimeter using a metal caliber, and the isolates classified as susceptible, intermediate and resistant (18). An isolate was considered multi-drug resistant (MDR) when it was found resistant for three and above different class of antibiotics.

**Quality assurance**: The reliability of our test procedures was ensured by implementing quality control measures throughout the whole process of the bacteriology lab work. Staining reagents, culture media, and antibiotic discs were checked for their normal shelf-life before use. All culture plates and antibiotic discs were stored at recommended refrigeration temperature (2–8°C) after preparation. Reference strains of *S. aureus* ATCC 25923, *E. coli* ATCC 25922, and *P. aeruginosa* ATCC 27853 were used as controls.

**Data analysis**: Data were entered by using Epidata version 3.2. and exported to the Statistical Package for the Social Science (SPSS) version 20 statistical software for analysis. Generated data were compiled by frequency tables and figures and other statistical summary measures. Logistic regression model was used to find factors associated with culture positive UTI and statistical significance was set at *p*<0.05.

**Ethical considerations**: The study was conducted after securing ethical approval of the protocol by institutional review (IRB) of college of medicine and health science, Bahir Dar University. Moreover, prior to commencing the study, a written informed consent/or assent was obtained from children and/or guardians. Subject confidentiality and any other special data security requirements were maintained and assured. Bacteriological positive results of pediatrics were communicated with health professionals for better management.

## Results

**Socio-demographic and clinical characteristics**: A total of 299 pediatric patients were included in this study. The mean age (with standard deviation) of the study participant was 6.6±5.5 years. Majority of the participants at 173 (57.9%), 154 (51.5 %) and 185 (61.9%) were females, urban residents and preschool children (0–7years), respectively. On top of this, most of the study participants had fever 211(70.6) and abdominal pain 178(59.5) (Table 1).

**Table 1:** Socio-demographic and clinical characteristics of the study participants at FHCSH and SBSPC (February 2020 to Jun 2020). N=299

	Variables	Frequency	Percent
Sex	Male	126	42.1
	Female	173	57.9
Residence	Urban	154	51.5
	Rural	145	48.5
Age	0–2	96	32.1
	3–6	89	29.8
	7–12	58	19.4
	13–18	56	18.7
Educational status	Pre-school	173	57.9
	School	126	42.1
Fever	Yes	211	70.6
	No	88	29.4
Vomiting	Yes	146	48.8
	No	153	51.2
Circumcision	Yes	85	28.4
	No	41	13.7
Dysuria	Yes	143	47.8
	No	156	52.2
Abdominal pain	Yes	178	59.5
	No	121	40.5
Frequent urination	Yes	130	43.5
	No	169	56.5
Flank pain	Yes	108	36.1
	No	191	63.9
History of UTI	Yes	42	14
	No	257	86
History of Catheterization	Yes	4	1.3
	No	295	98.7
Diabetes mellitus	Yes	27	5.7
	No	282	94.3
Constipation	Yes	82	27.4
	No	217	72.6
MUAC*	<13	43	14.4
	>13	246	85.6
History of discontinue antimicrobials treatment			
	Yes	50	16.7
	No	249	83.3

* MUAC: Mid-upper arm circumference

**Figure 1:**
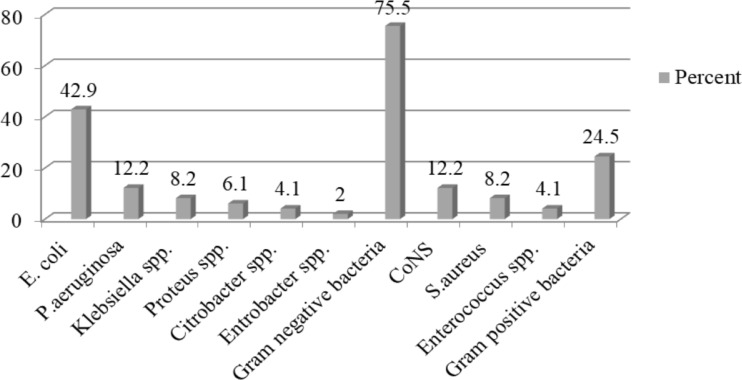
Percentage distribution of the isolates from pediatric patients with SBU at FHCSH and SBSPC (February–June 2020)

**Distribution of the isolates**: In this study the proportion of SBU was at 49 (16.4%). A total of 49 bacterial uropathogens were identified. Out of this, 12(24.5%) were Gram-positive and 37 (75.5%) were Gram-negative. *E. coli* at 21 (42.9%) was the most frequently isolated uropathogen followed by *P.aeruginosa* 6 (12.2%) and *CoNS*, 6 (12.2%). Among Grampositive isolates, *CoNS at* 6 (12.2%) was predominant followed by *S. aureus* 4 (8.2%) (Figure 1).

**Antimicrobial resistance profile of the isolates**: Both Gram-negative and Gram-positive isolates were tested for 11 different kinds of antibiotic discs as outlined in Table 2 and Table 3. Relatively high level of resistance was observed for ampicillin (97.3%), pipracllin (97.3 %) and amoxicillin-clavulnate (91.9%) by Gram-negative isolates. In contrast, these isolates were found sensitive for ciprofloxacin at (91.9%), meropenem (89%), ceftazidime (78.3%) and ceftriaxone (51.3%). *E. coli*, the most frequently isolated bacteria, was highly resistant for ampicillin and pipracllin. Furthermore, more than 78.4% of Gramnegative isolates showed multiple drug resistances. Similarly, Gram-positive isolates showed high level of resistance for ampicillin, 10/12 (83.3%) penicillin, 10/12(83.3%) and amoxicillin-clavulnate, 7/4(58.3%) and the level of multiple drug resistances of these isolates was at 50%. Generally, in this study the overall multi-drug resistance rate was at 71.4 % (Table 4).

**Table 2:** Antimicrobial resistance profile of Gram-positive isolates at FHRH and SBSPC (February – Jun, 2020)

Antibiotics tested	Number (%) of isolates			
	
	*S. aureus* (N=4)	*CoNS* (N=6)	*Enterococcus Spp.* (N=2)	Total (N =12)
AMP	4(100)	6(100)	0	10(83.3)
P	4(100)	6(100)	0	10(83.3)
OX	3(75)	ND	ND	3(75)
AMC	2(50)	4(66.7)	0	7(58.3)
SXT	0	4(66.7)	2(100)	6(50)
NIT	0	4(66.7)	0	4(33.3)
CHL	2(50)	4(66.7)	0	6(50)
CD	1(50)	2(33.3)	0	3 (25)
E	2(50)	1(16.7)	1(50)	4(33.3)
CIP	0	0	1(50)	1(8.3)
VAN	1(25)	2(33.3)	0	1(25)

**Abbreviations:** AMP, ampicillin; P, penicillin; OX, oxacillin; AMC, amoxicillin-clavulanate; SXT, trimehophrimsulfamethoxazol; NIT, nitrofurantoin; CHL, clindamycin; CD, chloramphenicol; E, erythromycin; CIP, ciprofloxacin; VAN, vancomycin. ND: not done.

**Table 3:** Antimicrobial resistance profile of Gram-negative isolates at FHRH and SBSPC (February – Jun, 2020)

Antibiotics tested	N (%) of the isolates						

	*E. coli* (n=21)	*P.aeruginosa* (n=6)	*Klebsiella* spp. (n=4)	*Proteus spp.* (n=3)	*Citrobacter* *spp.* (n=2)	*Entrobacter* *spp.*(n=1)	Total (n=37)
**AMP**	21(100)	6(100)	4(100)	3(100)	1(50)	1(100)	6(97.3)
**PIP**	21(100)	6(100)	3(75)	3(100)	2(100)	1(100)	36(97.3)
**AMC**	20(95.2)	5(83.3)	3(75)	3(100)	2(100)	1(100)	34(91.9)
**SXT**	15(74.4)	4(66.7)	3(75)	3(100)	1(50)	1(100)	22(59.5)
**CN**	13(61.9)	2(33.3)	3(75)	1(33.3)	0(0)	1(100)	20(54.1)
**NAL**	9(42.9)	ND	3(75)	2(66.7)	1(50)	1(100)	16(51.6)
**NIT**	12(57.1)	4(66.7)	3(75)	0	1(50)	1(100)	17(46)
**CRO**	8(38.1)	2(32.3)	2(50)	1(33.3)	1(50)	1(100)	15(45.6)
**CAZ**	5(23.8)	1(16.7)	1(25)	0	0	0	7(19)
**CIP**	0	1(16.7)	0	0	0	0	1(2.7)
**MR**	1(4.8)	1(16.7)	0	0	0	0	2(5.5)

CN, gentamicin; NAL, nialdxic acid; CRO, ceftriaxone; MR, meropnem

**Table 4:** multi-drug resistance profile of uropathogen identified from pediatric patients at FHCSH and SBSPC (February 2020 to June 2020)

List of antimicrobials	Gram Negatives isolates						Total

	*E. coli* *(n=21)*	*P.aeroginosa* *(n=6)*	*Klebsiella* *Spp, (n=4)*	*Proteus* *Spp(n=3)*	*Citrobacter* *spp, (n=2)*	*Entrobacter* *Spp, (n=1)*	
AMC, SXT, PIP, NAL					1		1
AMP, AMP, PIP, CN, SXT	2						2
AMP, CRO, AMC, SXT, PIP,	2	1					3
AMP, AMC, SXT, PIP, NIT		1		1			2
AMP, CN, AMC, NIT, PIP,	1	1			1		3
AMP, CN, AMC, SXT, PIP, NIT	1						1
AMP, CN, AMC, PIP,	3						3
NAT, CRO, CAZ							
AMP, CN, AMC, SXT, PIP,		1					1
CRO, NIT							
AMP, CN, AMC, SXT, PIP,	2		1				3
NAL, NIT							
AMP, CN, AMC, SXT, PIP,				1			1
NAL, CRO							
AMP, CN, AMC, SXT, PIP, NAL,			1				1
NIT, CRO							
AMP, CN, AMC, SXT, PIP,			1			1	2
NIT, NAL, CRO, CAZ							
AMP, CN, AMC, SXT, PIP, NAL,	4						4
NAT, CZN							
AMP, CN, AMC, SXT, PIP,		1					1
NAT, CZN, CIP, MR							
AMP, CN, AMC, SXT, PIP, NAT,	1						1
CRO, CZN, MR							
**No MDR detected**	5	1	1	1			8
**Total**	21	6	4	3	2	1	37

**Gram Positives isolates**				
	*S. aureus (n=4)*	CNS (n=6)	*Enterococcus spp* (n=2)	Total
SXT, E, CIP			1	
AMP, P, OX, AMC, E	1			1
AMP, P, OX, AMC, E, CHL, E	1			1
AMP, P, OX, E, CHL, CD, VAN	1			1
AMP, P, AMC, SXT, NIT, E, CHL, CD,		1		1
**AMP, P, AMC, SXT, NIT, CHL, CD, VAN**		1		1
**No MDR detected**	1	4	1	6
**Total**	4	6	2	12

**Factors associated with significant bacteriuria**: Based on the multivariable logistic regression analysis, females were about 12 times more likely to develop SBU than males (AOR: 12.014 (95%CI: 1.483–97.344) p= 0.020,). Similarly, circumcision status (for boys) (AOR: 0.063, (95%CI: 0.012–0.316) p= 0.001,), having flank pain (AOR: 18.077 (95%CI: 3.190–102.453) p= 0.001,) and being malnourished (AOR: 9.546, (95%CI: 1.834–49.687) p= 0.007,) were statistically associated with SBU (Table 5).

**Table 5:** Factors associated with significant bacteriuria at FHCSH and SBSPC (February–June 2020)

Variables		Significant Bacteriuria					
		
		Yes N (%)	No N (%)	COR	AOR	95% CI	p-value
Sex	Female	37(12.4)	136(45)	2.585	12.014		
	Male	12(4)	114(38.1)	1	1	1.483–97.344	0.020
Malnutrition	Yes	14(4,7)	29(9,7)	3.048	9.546		
	No	35(11,7)	221(73,9)	1	1	1.834–49.687	0.007
Circumcision	Yes	4(3.1)	81(65,6)	6.935	0.063		
	No	10(7.6)	31(23,7)	1	1	0.012–0.316	0.001
Flank Pain	Yes	34(11,4)	74(24.7)	5.391	18.077		
	No	15(5)	176(58.9)	1	1	3.190–102.453	0.001

**COR**-Crud Odds Ratio, AOR- Adjusted Odd Ratio, CI- Confidence Interval for the AOR

## Discussion

Urinary tract infection among the pediatric group is a significant source of morbidity and mortality around the globe. Pediatric patients with UTI require microbiologic investigation and proper treatment to minimize future complications. The prevalence of pediatric SBU in our study was at 16.4% (49/299). This finding is comparable with the previous studies conducted in India, Nepal and Ethiopia at 18.2%, 16% and 15.9% (20–22), respectively. Belete et al. and Fenta et al. had also reported 15.8% and 16.7% proportion of SBU among children at Felege Hiwot hospital in 2013 and 2019, respectively (23, 24). A lower prevalence was reported in Nigeria 3% (10). In contrast, higher proportion of SBU among the pediatric group was also reported in Nairobi, Kenya, 69.7% (25), Congo, 42.2% (26) and in Ethiopia at (26.5–27.5%) (27, 17).

The variation of the reported prevalence of SBU among the pediatric population across different studies from one country to other or among region of the same country might be attributed to the difference in sample size, geographical variations, host factors and social habit of the community and health education practice, environmental conditions and the standard of personal hygiene.

In the present study, Gram-negative isolates were predominant with a proportion at 75.5% which is in agreement with similar studies done in Ethiopia 82.4%–82.9% (17, 27). This finding supports the fact that Gram-negative bacteria are the most predominant uropathogens that usually sourced from the bowel and ascend to the urinary tract. They have also unique structures (like, pilus adhesions) which help the bacteria for attachment to the uroepithelium lining and prevent them from urinary lavage, allowing for multiplication and tissue invasion resulting in invasive infections (4).

*E. coli* at 21 (42.9%) was the most frequent isolate followed by *P. aeruginosa*, 6 (12.2%) and *CoNS*, 6 (12.2%). This is in agreement with other studies somewhere in the world (20, 28, 29) and in Ethiopia (21). *E. coli* is the commonest flora of the gastrointestinal tract from which it ascends to urinary tract and it has well characterized virulence factors for colonization.

With regard to AMR profile of the isolates, in the present study, relatively high level of resistance was observed for ampicillin (97.3%), pipracillin (97.3 %) and amoxicillin-clavulanate, (91.9%) by Gram-negative isolates. However, these isolates showed better sensitivity for ciprofloxacin at (91.9%), meropnem (89%), and ceftazidime (78.3%).

Our finding is in agreement with previous reports from Tanzania (29) and Ethiopia (27). Specifically, *E. coli*, showed 100%, 95.2% and 74.4%, resistant for ampicillin, pipracillin, and amoxicillin-clavulanate, respectively. The level of AMR stated above might be attributed by a number of factors including over and misuse of antimicrobials in the study area where there is weak regulatory practice and inadequate bacteriological surveillance due to lack of routine antimicrobial susceptibility testing facilities. Most of the antimicrobials listed are available without prescription in local pharmacies and people could purchase and use them without prescription. This would also play its big share for high level antimicrobial resistance reported in this study.

Gram-positive isolates showed high level of resistance for ampicillin, 10/12 (83.3%) penicillin, 10/12(83.3%) and amoxicillin-clavulanate, 3/4(75%). However, better sensitivity was documented for ciprofloxacin, vancomicin and clindamycin, at (91.7%), (75%) and (58.3%), respectively. A study in other parts of Ethiopia reported comparable finding with our report (17).

The overall Multidrug resistance (MDR) rate in this study was at 71.4%. Specifically, 78.4% and 50% of Gram-negative and Gram-positive isolates, respectively showed MDR. Similar studies conducted in Ethiopia showed 58.3%–73.7% (17, 21) level MDR uropathogens among the pediatric group. As we described above, this high level of MDR might be due to the wide spread and misuse of antibiotics, inappropriate prescription of drugs and lack of knowledge about drug resistance in the study area.

Concerning factors associated with SBU; in this study sex, circumcision status, having a flank pain and being malnourished were statistically associated with SBU (*p*< 0.05). Females were about 12 times more likely to have SBU than males (AOR = 12.014, P= 0.020, CI = 1.483 - 97.344). The higher rate of SBU in females might be as a result of their shorter urethra and the proximity of their reproductive organ to the anus. This finding is supported by other studies in Ethiopia (17, 21, 27).

Generally, this study revealed high prevalence of significant bacteriuria among the pediatric group in the study area. *E. coli* was the most predominant isolate followed by *P. aeruginosa* and *CoNS*. A large number of the isolates were found resistant to the commonly prescribed antimicrobials in the study area. The overall MDR rate in this study was higher. Relatively low level of resistances was documented against ciprofloxacin, meopenem, ceftazidime, ceftriaxone and vancomycin. Hence, coupled with other similar study reports, these drugs could be considered as an empirical therapy for UTI for pediatric patients. Sex, circumcision status, having a flank pain and being malnourished were statistically associated with SBU (*p*< 0.05).

Therefore, continuous health education, rational use of antimicrobials, collaborative regular surveillance of pathogens associated with UTI with their antimicrobial resistance pattern should be done to reduce the magnitude of SBU among the pediatric population. Empirical antibiotic selection should be based on the knowledge of local prevalence of bacterial organisms and their updated antibiotic susceptibility patterns rather than on the universal guidelines. Further study should be considered to molecularly characterize the isolates and to identify bacteriological factors associated with multiple drug resistance.

This study provided important data on the type of bacterial isolates, their AMR profile and factors associated with SBU among the pediatric group. However, our study has some confines that should be considered while interpreting the finding. We did not attempt to identify nonbacterial uropathogens and few bacterial isolates were not identified at a species level and no serotyping was also done for some isolates due to resource limitation. The other limitation was usage of Kirby-Bauer disc diffusion method to perform AST instead of micro-dilution method, which is more sensitive.
